# Sucralose activates an ERK1/2–ribosomal protein S6 signaling axis

**DOI:** 10.1002/2211-5463.12172

**Published:** 2017-01-18

**Authors:** Marcy L. Guerra, Michael A. Kalwat, Kathleen McGlynn, Melanie H. Cobb

**Affiliations:** ^1^ Department of PharmacologyUT Southwestern Medical CenterDallasTXUSA; ^2^ Present address: StemSynergy TherapeuticsNashvilleTNUSA

**Keywords:** insulin secretion, pancreatic islet beta cells, sucralose, sweet taste receptor

## Abstract

The sweetener sucralose can signal through its GPCR receptor to induce insulin secretion from pancreatic β cells, but the downstream signaling pathways involved are not well‐understood. Here we measure responses to sucralose, glucagon‐like peptide 1, and amino acids in MIN6 β cells. Our data suggest a signaling axis, whereby sucralose induces calcium and cAMP, activation of ERK1/2, and site‐specific phosphorylation of ribosomal protein S6. Interestingly, sucralose acted independently of mTORC1 or ribosomal S6 kinase (RSK). These results suggest that sweeteners like sucralose can influence β‐cell responses to secretagogues like glucose through metabolic as well as GPCR‐mediated pathways. Future investigation of novel sweet taste receptor signaling pathways in β cells will have implications for diabetes and other emergent fields involving these receptors.

**Abbreviations**
BRET: bioluminescence resonance energy transfer
CAMYEL: sensor for cAMP
ERK1/2: extracellular signal‐regulated kinases 1 and 2
GLP‐1: glucagon‐like peptide 1
GPCR: G protein‐coupled receptor
KRBH: Krebs–Ringer bicarbonate solution
RSK: ribosomal S6 kinase

Artificial sweeteners have been reported to induce insulin secretion from pancreatic β cells through the sweet taste receptor 1, 2, 3, 4, 5, 6, 7, a G protein‐coupled receptor (GPCR), a dimer of T1R2 and T1R3 taste receptor subunits 8. The T1R3 subunit can heterodimerize not only with T1R2 but also with at least one other class C GPCR subunit, T1R1, to form the umami taste receptor which can be activated by many l‐ and a few d‐amino acids 9. T1R3 has also been suggested to act as a homodimer to sense sweeteners 3, 10. The taste receptor subunits are members of GPCR class C with large Venus flytrap extracellular domains that have a range of abilities to bind sweet compounds, amino acids, and some other small molecules 11. The sweet taste receptor is widely expressed but was originally identified as a gustatory taste receptor linked to a sweet‐responsive locus in mice 12, 13, 14, 15. In addition to glycine and sweet‐tasting amino acids, the sweet taste receptor responds to fructose, other sugars, and a variety of artificial sweeteners 16, 17. Because rodent and human T1Rs are only ~70% identical in amino acid sequence, reported differences in responsiveness of the sweet taste receptor to specific compounds may represent species‐selective sensitivities. Mouse sweet taste receptors, for example, are activated by fructose but apparently not by glucose 2.

Initial studies on T1Rs focused on taste sensing by the tongue and concluded that taste receptors signal through the Gα_i_ family member α‐gustducin 18, 19, 20, 21. All three T1R subunits and α‐gustducin are present in mouse and human pancreatic islets and have been implicated in regulation of insulin secretion 1, 22, 23. Reconstitution of G protein‐coupled signaling using purified G proteins and purified seven transmembrane‐spanning core domains of T1R1, T1R2, and T1R3 showed that T1R1 and T1R2 activate Gα_i/o_ family members but not Gα_s_ or Gα_q_, while T1R3 was unable to activate any of the G_α_ subunits tested 24. Nevertheless, α‐gustducin knockout mice are still able to sense sweet and umami flavors, suggesting that other G proteins may also be able to initiate signals from taste receptors 25. More recently, these receptors were shown to be involved in sweet sensation in enteroendocrine L‐cells and in pancreatic β cells 1, 23, 26, 27, 28. In β cells, hormone‐dependent inhibition of insulin secretion often occurs through G_i_‐dependent mechanisms, suggesting that receptors activating insulin secretion, such as taste receptors, will not signal through G_i_, and, instead, activation of G_i_ will block their actions 29.

We previously found that amino acids activate the mitogen‐activated protein kinases ERK1/2 through the umami receptor T1R1/T1R3 in several cell types including the MIN6 β cell line 23. ERK1/2 activation in these cells depends on G_q_ and calcium signaling 28. Knockdown of the shared subunit T1R3 decreased insulin content and insulin secretion 23. The loss of insulin content and reduced secretion may have been due to impaired function of both the umami and the sweet taste receptor. Artificial sweeteners, sucralose, saccharin, and acesulfame‐K, are reported to induce insulin secretion from rodent islets and MIN6 β cells 1. The activity of sweeteners was blocked by gurmarin, a peptide inhibitor of rodent T1R1/T1R2 25, 30, providing evidence that sucralose and other sweeteners do indeed mediate their effects through a sweet receptor‐mediated mechanism. Fructose enhanced glucose‐induced but not basal insulin secretion in mice and isolated mouse and human islets 2. This was not the case in T1R2^−/−^ mice, islets isolated from these mice, or human islets treated with the T1R3 inhibitor lactisole. Used at higher concentrations, lactisole was shown to inhibit mouse T1R3 as well, and blocked glucose‐induced insulin secretion and sweetener responses in mouse islets, suggesting an alternate role for T1R3 homodimers in sweet sensing 31.

To delve into sweet receptor signaling in pancreatic β cells, we have examined effects of the sweet compound sucralose on insulin secretion, ERK1/2 activation, calcium influx, cAMP generation, and other downstream pathways. Sucralose serves as a tool to activate the sweet taste receptor without involving metabolism. We also compared sucralose and GLP‐1 effects, given their similar GPCR signaling pathways. Our data support a signaling cascade starting with sweetener activation of the sweet taste receptor and induction of calcium and cAMP increases, activation of ERK1/2, and leading to site‐specific phosphorylation of ribosomal protein S6. This pathway apparently acts independently of mTORC1 or RSK, leaving open the possibility of role for sucralose signaling through other ERK1/2‐dependent S6 kinases. We propose that sweet taste receptor signaling may be acting under glucose‐stimulated conditions and given its effects on ribosomal protein S6, we speculate an influence on translation. The implications of these results stem from the utility of sucralose to deconvolve sweet taste receptor signaling from metabolism, and suggest that sweet taste receptor signaling can influence β‐cell responses to secretagogues like glucose through metabolic as well as GPCR‐mediated pathways. Future investigation of novel sweet taste receptor signaling pathways in β‐cells will have implications for diabetes and other developing fields in which these receptors are being identified.

## Materials and methods

### Materials

The following materials were obtained from the indicated vendors: Fura‐2 AM from Molecular Probes (Eugene, OR, USA); d‐fructose and sucralose from Sigma (St. Louis, MO, USA). All other reagents were obtained through Fisher Scientific (Waltham, MA, USA) unless otherwise stated.

### Cell culture

MIN6 cells were cultured as described 23. Unless otherwise stated, MIN6 cells were preincubated for 3 h in Krebs–Ringer bicarbonate solution (KRBH) (115 mm NaCl, 5 mm KCl, 24 mm NaHCO_3_, 1 mm MgCl_2_, 2.5 mm CaCl_2_, 25 mm HEPES pH 7.4, 0.1% BSA) with 4.5 mm glucose prior to stimulations.

### Calcium assays

Assays were as previously described 32. Cells were plated in white‐walled 96‐well plates (Costar 3903) in a final volume of 0.2 mL per well. To prepare for calcium influx assays, cells were washed twice with PBS, loaded for 1 h with Fura‐2AM (5 μm) in KRBH containing 4.5 mm glucose, and then washed twice more to remove excess Fura‐2AM. After 30 min of de‐esterification, agents were added to triplicate wells using injectors. Changes in ${\text{Ca}}_{i}^{2+}$ were assessed every 0.74 s by dual excitation of Fura‐2 at 340/11 and 380/20 nm (center/bandpass) and emission at 508/20 nm with a SynergyJ2 multi‐mode microplate reader (BioTek, Winooski, VT, USA), gen5j software. For experiments in which reads were for 1 h, cells were incubated in KRBH with glucose as indicated for 3 h totally (1.5 h during and prior to Fura‐2AM loading/equilibration) prestimulation with agents. Agents (2X concentrated) were applied manually using a multichannel pipette to triplicate wells in equal volumes as the preincubation buffer. Fura‐2 fluorescence was monitored every 10 s for 1 min prior to stimulation and every 10 sec for 1 h poststimulation. To determine changes in free intracellular Ca^2+^, the basal 340/380 ratio was averaged before and after stimulation. The average value was subtracted from the pre‐ and poststimulation values.

### Detection of cAMP using an intracellular cAMP sensor

The Epac‐based bioluminescence resonance energy transfer (BRET) sensor for cAMP (CAMYEL) has been described 33. MIN6 cells were infected with a retrovirus expressing CAMYEL and selected for stable expression using G418. BRET assays were performed on the microplate reader as above. Emission signals at 485/20 and 528/20 nm (center/bandpass) were measured every 0.8 s for 1 min (basal) and for 4 min after stimulation.

### Immunoblotting and immunoprecipitation kinase assays

Cells were lysed in 50 mm HEPES (pH 7.5), 150 mm NaCl, 1% Triton X‐100, 10 μg·mL^−1^ aprotinin, 5 μg·mL^−1^ leupeptin, 5 μg·mL^−1^ pepstatin A, 0.2 mg·mL^−1^ PMSF, 100 mm NaF, and 2 mm Na_3_VO_4_. Lysates were stored at −80 °C and cleared by sedimentation at 16 000 ***g*** for 10 min at 4 °C prior to analysis. Lysate protein (40 μg) was resolved by SDS/PAGE and transferred to nitrocellulose membranes. Membranes were blocked with 5% nonfat milk in Tris‐buffered saline containing 0.1% Tween‐20 (TBST) for 2 h and incubated with primary antibodies overnight at 4 °C. Antibodies were diluted in 5% milk/TBST as follows: pERK1/2 (1 : 1000, Sigma, #M8159, mouse, monoclonal), ERK1/2 (1 : 3000, Y691 rabbit 32). Antibodies to pS6K (T389) (1 : 1000, #9206L), S6 (1 : 500, #2317S), pS6 (S235/236) (1 : 1000, #2211S), and pS6 (S240/244) (1 : 2000, #5364S), pp70S6K (Thr389) (1 : 1000, #9206), phospho‐p38 (1 : 250, #9211), and phospho‐JNK1/2 (1 : 250, #9255) were from Cell Signaling (Danvers, MA, USA). Antibodies to p70S6K (1 : 250, sc‐230), p38 (1 : 1000, sc‐7972), and JNK1/2 (1 : 4000, sc‐571) were from Santa Cruz (Dallas, TX, USA). Membranes were washed with TBST and incubated for 1 h with secondary antibodies: Donkey anti‐rabbit IRDye 680RD or donkey anti‐mouse IRDye 800CW (1 : 10 000, LI‐COR Biosciences, Lincoln, NE, USA). Membranes were washed with TBST, imaged and quantified using the LI‐COR Odyssey Infrared imaging system. Immunoprecipitation of p70‐S6K and subsequent kinase assays were performed as previously described 34.

### Insulin secretion

MIN6 cells seeded in 24‐well plates were washed once with PBS and preincubated with KRBH (1 mL per well) for 2 h. The buffer was then removed and treatments made in KRBH were added (1 mL per well). Treatment buffer was then collected after 30 min and cleared by centrifugation at 2000 ***g*** for 2 min. Cells were lysed in 200 μL lysis buffer per well and protein was quantified (as described for immunoblotting). Secreted insulin values were quantified using the Mercodia Insulin ELISA Assay and normalized to protein. Data are expressed as fold over basal (KRBH alone) treatment values (ng insulin secreted per mg protein).

### RNA isolation, cDNA synthesis, and real‐time quantitative PCR

Cells or human islets were harvested in TRI Reagent Solution and RNA was extracted as per the manufacturer's instructions. Human islets were provided by the Integrated Islet Distribution Program. cDNA was generated using the High Capacity cDNA Reverse Transcription kit (Applied Biosystems, Foster City, CA, USA). cDNA was used for RT‐PCR of T1R2, and 18S was used as an internal expression control. The primers were as follows: 18S forward, 5′‐CGCGGTTCTATTTTGTTGGT‐3′ and reverse, 5′‐AGTCGGCATCGTTTATGGTC‐3′; T1R2 forward, 5′‐TCATCACCCTCAGCATGACCTTCT‐3′ and reverse, 5′‐CTCCGGGTAGAAAAGGATCATGTA‐3′.

### Statistical analysis

Results are expressed as the mean ± SE determined from at least three independent experiments. Comparisons were made using Student's *t*‐test and were considered statistically significant at *P* < 0.05.

## Results

### Sucralose stimulates insulin secretion, cAMP, and calcium influx in MIN6 beta cells

**Figure 1:**
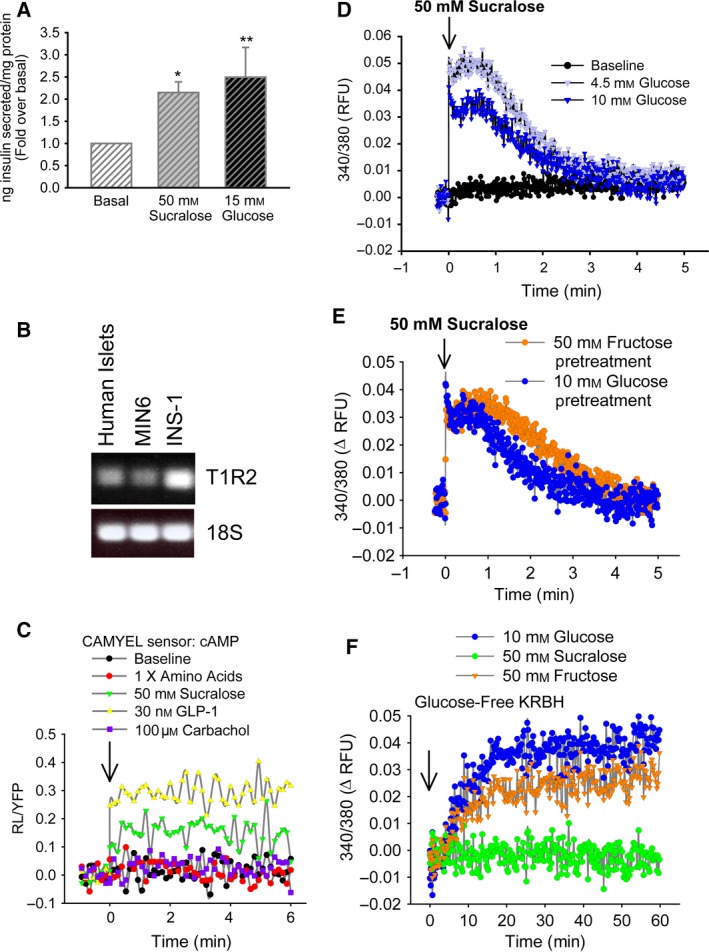
Sucralose signaling in MIN6 beta cells induces insulin secretion, cAMP elevation, and nutrient‐dependent calcium influx. (A) Sucralose and glucose stimulation for 30 min causes insulin secretion in MIN6 cells. Basal is 4.5 mm glucose. *P < 0.05 vs. basal. (B) RT‐PCR shows presence of T1R2 transcript in human islets, MIN6, and INS‐1 β cells. (C) Intracellular cAMP was measured in MIN6 cells stably expressing the CAMYEL BRET sensor. Cells in KRBH were stimulated with AA (*n* = 4) or GLP‐1 (*n* = 5). (D) Sucralose activates calcium influx in MIN6 cells only in the presence of glucose. (E) Sucralose‐induced calcium influx in MIN6 cells in the presence of glucose or fructose. (F) Calcium influx initiated by glucose, sucralose, or fructose under glucose‐free basal conditions in MIN6 cells. Sucralose has no effect on calcium influx in glucose‐free conditions. Data were analyzed as in Materials and methods. Data are the mean ± SE. *P < 0.05 vs basal.

To examine effects of sweeteners on insulin secretion, we compared insulin secretion from MIN6 cells in basal 4.5 mm glucose, stimulated with 15 mm glucose or 50 mm sucralose for 30 min. Confirming previous reports 1, the increase in insulin secretion caused by 50 mm sucralose was similar to that caused by glucose (Fig. 1A). Sucralose is known to signal through the sweet taste receptor, T1R2, and in support of this we detected expression of the T1R2 mRNA transcript in human islets, MIN6 and INS1 β cells (Fig. 1B). Sucralose had also been reported to induce changes in cAMP in MIN6 cells. We examined effects of sucralose and amino acids on cAMP compared to glucagon‐like peptide 1 (GLP‐1), which works through Gs, and the M3 muscarinic receptor ligand carbachol, which activates Gq and does not increase cAMP. Sucralose clearly increased cAMP in the cells within 1 min and sustained the elevation over the 6‐min time course, but to a lesser extent than GLP‐1 (Fig. 1C). Neither amino acids nor carbachol had any effect on cAMP (Fig. 1C). Increases in intracellular calcium were also induced by sucralose (Fig. 1D); however, sucralose‐induced changes were nutrient‐dependent as no increase was observed without glucose or fructose (Fig. 1D,E). The requirement for glucose was also noted for stimulation by amino acids 28. Fructose or glucose stimulation of cells preincubated without glucose caused increased intracellular free calcium (Fig. 1F), consistent with their ability to be metabolized, while sucralose had no effect.

### Sucralose‐induced signaling is nutrient‐dependent

**Figure 2:**
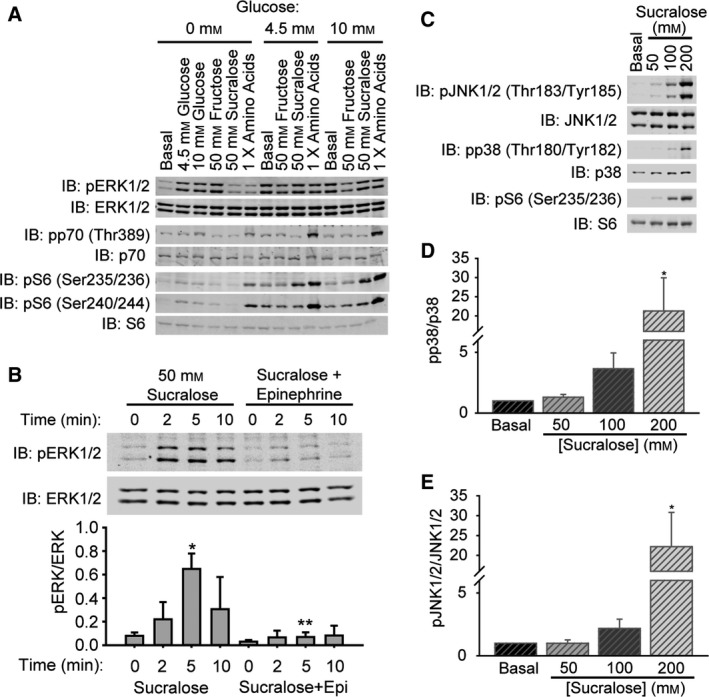
Sucralose activation of ERK1/2 requires glucose and is inhibited by epinephrine in β cells. (A) Glucose‐dependent activation of ERK1/2 and S6 by sucralose and amino acids in MIN6 cells. Cells were preincubated in 0, 4.5, or 10 mm glucose followed by stimulation with indicated agents for 30 min. (B) Sucralose stimulation of MIN6 cells increases pERK1/2 by 2 min and is blocked by pretreatment with epinephrine (10 μm). *P < 0.05 0 min vs 5 min sucralose, **P < 0.05 5 min sucralose vs 5 min sucralose+epinephrine. (C) Sucralose (50 mm) does not cause S6 phosphorylation due to osmotic stress. Dose–response of sucralose stimulation for 30 min of MIN6 cells. Activation of JNK1/2 and p38 was monitored as reporters of osmotic stress. Relative quantitation of p38 (D) and JNK1/2 (E) activation is displayed as bar graphs. *P < 0.05.

Extracellular signal‐regulated kinases 1 and 2 activation is a rapid indicator of the demand for insulin secretion in response to nutrients, hormones and neurotransmitters, and leads to nutrient‐dependent insulin gene transcription 35. Therefore, we tested effects of sucralose on ERK1/2. Sucralose rapidly activated ERK1/2 in MIN6 cells preincubated in 4.5 mm glucose (Fig. 2A), as previously reported 1. Neither sucralose nor amino acids activated ERK1/2 in cells preincubated without glucose; on the other hand, 50 mm fructose increased ERK1/2 activity almost as well as glucose (Fig. 2A). Insulin secretion and activation of ERK1/2 by glucose, GLP‐1, and other agents that induce insulin secretion is blocked by α_2_‐adrenergic agonists such as epinephrine that work through Gi 28. Sucralose‐stimulated ERK1/2 activation was also prevented by preincubation with epinephrine (Fig. 2B). These results parallel the glucose requirement for sucrose to elevate intracellular calcium and are expected for amplifiers of insulin secretion.

Previously, we found that amino acids stimulated mTORC1 in MIN6 cells through T1R1/T1R3 and that the increase in mTORC1 activity, measured as increased phosphorylation of the mTORC1 substrate p70 S6 kinase, also required glucose 23, 28. Amino acids were tested as a control (Fig. 2A), which shows that an amino acid‐induced increase in phosphorylation of p70 on Thr 389 and of ribosomal protein S6 is observed in 4.5 or 10 mm glucose, but not without glucose. Glucose, sucralose, and fructose had little or no effect on phosphorylation of p70, but did promote phosphorylation of the p70 substrate S6. In particular, sucralose promoted phosphorylation of S6 at Ser235/236 (Fig. 2A), sites often phosphorylated by kinases other than p70. To verify that sucralose‐induced signaling was specific and not due to stress, we analyzed c‐Jun N‐terminal kinase (JNK) and p38 MAP kinase activities and found that only 100 or 200 mm doses of sucralose significantly impacted these stress‐responsive pathways (Fig. 2C–E).

### S6 Ser 235/236 activation by sucralose is ERK1/2 pathway‐dependent

**Figure 3:**
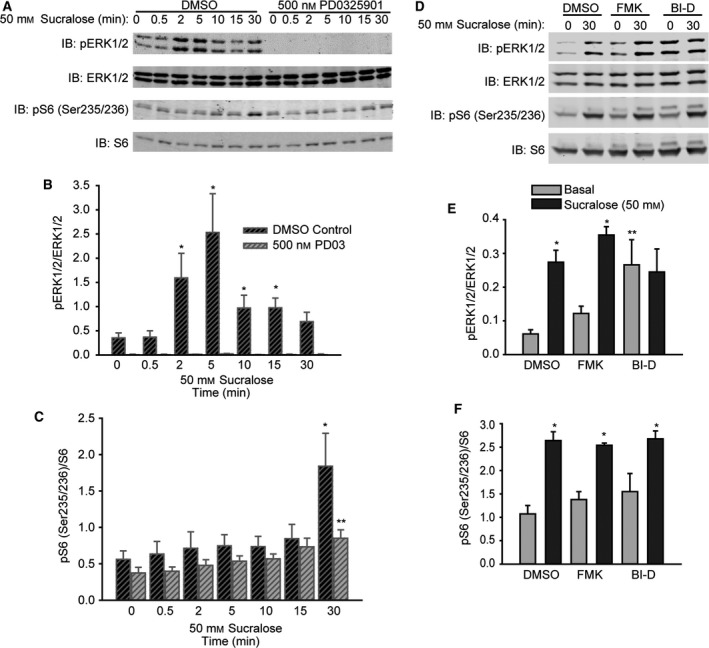
Sucralose‐induced signaling promotes S6 Ser 235/236 phosphorylation in an ERK1/2‐depenedent manner. (A–C) Sucralose activated ERK1/2 at 2–5 min and S6 pSer 235/236 at 30 min. Both events were abolished by pretreatment with the MEK1/2 inhibitor PD0325901 (A). Quantitation of immunoblots for pERK1/2/ERK1/2 (B) and S6 pSer 235/236 (C). Bar graph data are the mean ± SE. *P < 0.05 vs 0 min. **P < 0.05 vs DMSO control. (D–F) Pretreatment of MIN6 cells with RSK inhibitors FMK (2 μm) or BI‐D1870 (100 nm) (BI‐D) does not prevent sucralose‐induced S6 and ERK1/2 phosphorylation (D). Quantitation of immunoblots for pERK1/2/ERK1/2 (E) and S6 pSer 235/236 (F). Bar graph data are the mean ± SE. *P < 0.05 vs Basal. **P < 0.05 vs DMSO control.

To investigate the site‐specific activation of S6 by sucralose, we examined the effect of the ERK pathway on S6 phosphorylation by inhibiting the upstream kinase of ERK, MEK. The MEK inhibitor blocked ERK activation by sucralose in 4.5 mm glucose over the 30‐min time course (Fig. 3A,B). In addition to p70, several other kinases can phosphorylate S6 including cyclic AMP‐dependent protein kinase (PKA), casein kinase I (CKI), and the ERK1/2 substrates ribosomal protein S6 kinases (Rsks) 36, 37, 38, 39, 40, 41, 42. Preventing sucralose‐induced ERK activation significantly blunted S6 Ser 235/236 phosphorylation at 30 min (Fig. 3C). Pretreatment with known inhibitors of Rsk with distinct mechanisms suggested that sucralose did not require Rsk to cause phosphorylation of either ERK1/2 or S6 (Fig. 3D–F). BI‐D1870 raised basal ERK1/2 activation even at relatively low doses (Fig. 3D,E), although it did not impact basal S6 phosphorylation (Fig. 3F). These results support a previously unknown sweetener‐induced ERK1/2 pathway leading to S6 phosphorylation, an event commonly linked to growth factors and nutrients.

### Sucralose‐induced S6 Ser 235/236 phosphorylation is independent of mTORC1

**Figure 4:**
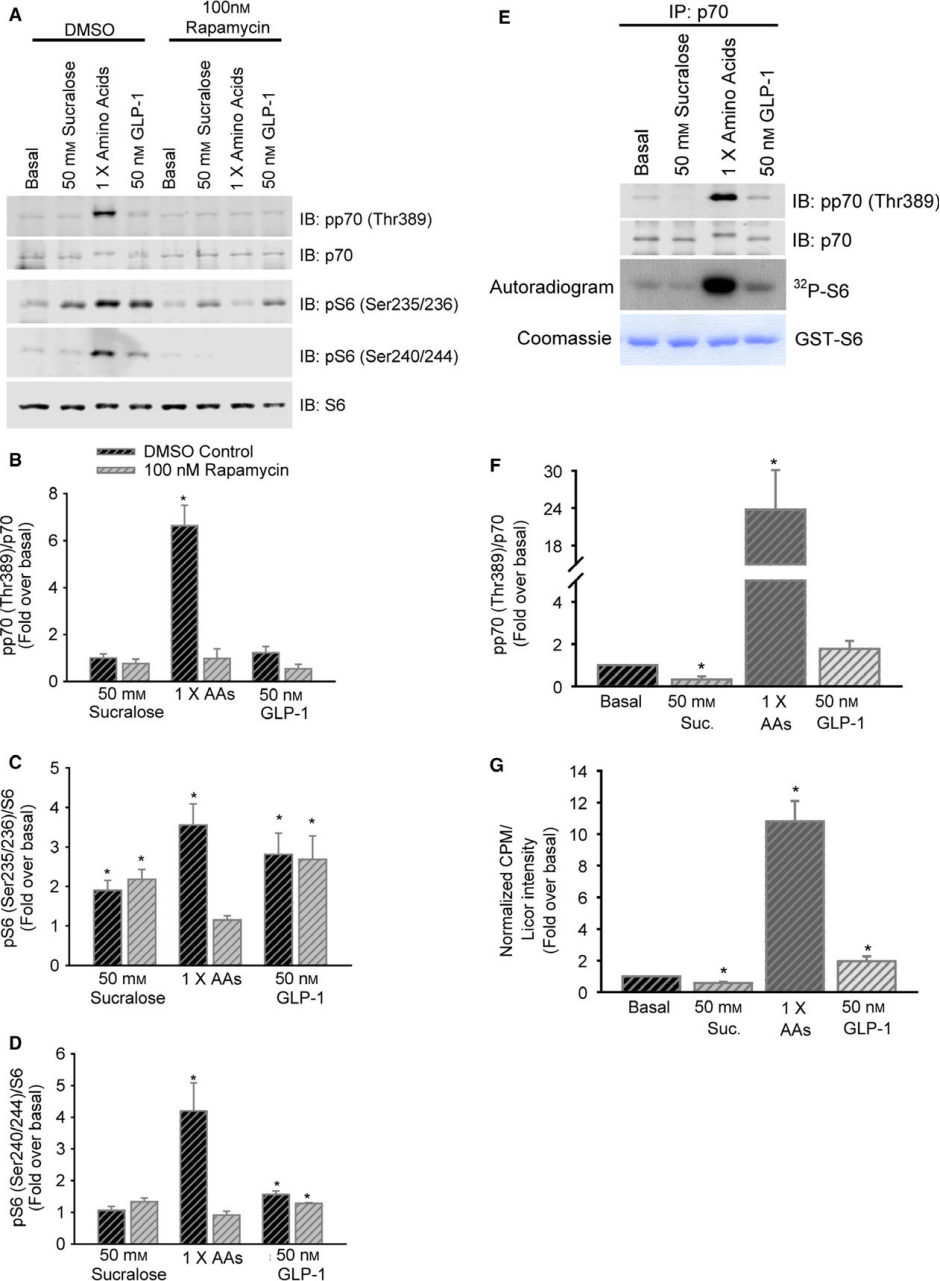
Sucralose activation of S6 is mTOR pathway‐independent. (A) MIN6 cells stimulated with sucralose, amino acids, or GLP‐1 in the absence or presence of rapamycin. (B–D) Relative quantitation of immunoblots from (A) for activation of p70 (B), S6 Ser 235/236 (C), and S6 Ser 240/244 (D). *P < 0.05 vs Basal. (E) MIN6 cells were treated with sucralose, amino acids, or GLP‐1, and p70 was immunoprecipitated. Kinase assay using GST‐S6 shows amino acids robustly activate p70, while sucralose does not. Relative quantitation of p70 activation by immunoblotting (F) and normalized radiolabel incorporation into GST‐S6 (G) is displayed as bar graphs of the mean ± SE. *P < 0.05 vs Basal.

**Figure 5:**
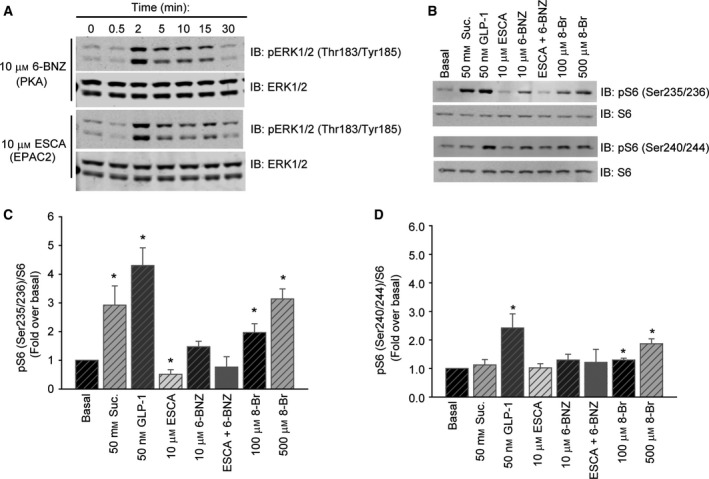
Sucralose, GLP‐1, and cAMP analogs each induce S6 phosphorylation at Ser235/236. (A) MIN6 cells were stimulated with either 6‐BNZ or ESCA to activate PKA or EPAC, respectively for the indicated times. (B) MIN6 cells stimulated with sucralose, GLP‐1, or combinations of PKA and EPAC activators. S6 Ser 235/236 and Ser 240/244 activation was monitored by immunoblotting. Relative quantitation of the phospho‐S6 immunoblots are shown in (C) and (D). Bar graphs are the mean ± SE. *P < 0.05 vs Basal.

To determine whether mTORC1 and p70 contribute to the effects of sucralose, we compared the ability of sucralose, amino acids, and GLP‐1 to induce phosphorylation of p70 and S6 in the presence of the mTORC1 inhibitor rapamycin. The positive control, amino acids, activated p70 and S6 phosphorylation through mTORC1 (Fig. 4A–D), as evidenced by rapamycin sensitivity and elevated p70 activity in an immunoprecipitation kinase assay (Fig. 4E–G). Sucralose did not activate p70 (Fig. 4A,B) and induced phosphorylation of S6 specifically on Ser 235/236 (Fig. 4A,C) that was rapamycin insensitive. Surprisingly, GLP‐1 activated mTORC1 to a small extent as shown by phosphorylation observed on Ser240/244 that was sensitive to rapamycin (Fig. 4A,D) and increased p70 activity in the immune‐complex kinase assay (Fig. 4E–G). We further evaluated the potential pathways utilized by sucralose and GLP‐1 during S6 phosphorylation by determining the extent to which cAMP‐stimulated effectors, both PKA and Epac 43, 44, 45, 46, 47, could differentially regulate S6 phosphorylation sites. We first validated that activators of PKA (6‐BNZ) or EPAC2 (ESCA) led to induction of ERK1/2 activation (Fig. 5A). While sucralose and GLP‐1 each induced S6 Ser 235/236 phosphorylation, ESCA apparently inhibited this event and 6‐BNZ slightly increased it (Fig. 5B,C). The nonspecific cAMP analog 8‐Br‐cAMP clearly induced S6 Ser 235/236 phosphorylation (Fig. 5B,C). S6 phosphorylation on Ser 240/244 was only induced by GLP‐1 and 8‐Br‐cAMP, although to a lesser extent than that of Ser 235/236 (Fig. 5B,D). The data show that sucralose and GLP‐1 are each able to induce S6 phosphorylation at Ser235/236 on their own. It remains to be determined whether sucralose acts on S6 via cAMP‐induced PKA activation 48.

## Discussion

Activation of T1R1/T1R3 and T1R2/T1R3 in taste cells has been shown to couple to the heterotrimeric G protein α‐gustducin, a Gα_i_ family member, and a cascade of events including Gβγ subunits, increased phospholipase (PLC) β‐2 activity, increased inositol triphosphate (IP_3_), intracellular Ca^2+^ release, transient receptor potential TRPM5 ion channels, and membrane depolarization 49. A similar pathway has been proposed in small intestine enteroendocrine L‐cells 50, 51, 52. Based on the distinct signaling pathways affected, inhibition by epinephrine, and earlier work on umami receptors 28, we conclude that the G protein wiring in β cells is distinct from that described in these other tissues. Inhibition of sucralose‐stimulated ERK1/2 activity by epinephrine likely indicates that Gi is blocking, not mediating, actions of sweet receptors in β cells, as is also the case for the umami receptor 28. Other recent findings in β cells suggest that Gα_s_ and Gα_q_ family members including Gα_14_ are important in taste receptor signaling 1, 28, 53, 54, consistent with our results. An explanation for differences in G protein utilization is likely that G protein signals coupled to these receptors are specialized to the functions of the cell type or tissue. For example, recent work indicates that sweet taste receptors alter innate immunity in the human upper respiratory system 55. This suggests a broader functional range for taste receptors than initially anticipated, and has been suggested to relate to allelic variation in these genes 56, 57.

Signaling events induced by sucralose in β cells include increased intracellular free calcium, cAMP, and phosphorylation of ERK1/2 and S6, but no increase in mTORC1 activity. Activation of the umami receptor by amino acids increases calcium, ERK1/2, and mTORC1 activities, but not cAMP. In both cases, the presence of glucose is required to activate ERK1/2. Activation of insulin secretion only if glucose is present is characteristic of many GPCRs that act as amplifiers or potentiators of insulin secretion (e.g., GLP‐1). These results add further weight to the conclusion that nutrient sensing occurs at the β cell plasma membrane and contributes to control of insulin production and secretion. With this in mind, sucralose is a useful *in vitro* tool to deconvolute the effects of sweet taste receptor signaling from glucose metabolism. An unanticipated finding was that GLP‐1 activated mTORC1 signaling, and also retained a rapamycin‐resistant arm of signaling to S6 Ser 235/236. This may relate to the growth‐promoting effects of GLP‐1 58, 59, 60 and suggests the involvement of both ERK‐RSK and mTORC1 pathways. Early work from the Thomas laboratory suggested that S6 phosphorylation is ordered, beginning with phosphorylation of S235/236 and continuing to more C‐terminal residues including phosphorylation of Ser 240/241 61. More recently, Roux and Blenis presented evidence that RSK selectively phosphorylates S6 on 235/236 but does not proceed to phosphorylate 240/244 62, 63. Our findings on phosphorylation of specific sites and sensitivity to MEK inhibition favor the idea that either RSK or a related enzyme is the relevant kinase, in spite of the poor congruence with inhibitor findings. Given that there are at least four distinct RSKs in mammalian cells, it is possible that the chemical inhibitors could not equally and specifically suppress the all of relevant RSK activity downstream of sucralose.

The consequences of activation of sweet receptors by artificial sweeteners in endocrine cells remain uncertain. Previously, the T1R3 inhibitor lactisole inhibited glucose‐induced GLP‐1 secretion from human L‐cells and was also reported to inhibit GLP‐1 secretion in human subjects after intragastric or intraduodenal glucose infusion 26, 64. Recent reviews summarize the disparities among studies and argue against direct effects of artificial sweeteners on islets 7, 65. Part of the discrepancy in the studies with human subjects could be related to common polymorphisms in the T1R1 and T1R3 genes, noted above, that affect the ability of these receptors to be activated by various ligands 56, 57, 66, 67. As glucose can also act through this receptor, but is also metabolized, it is difficult to deconvolve the metabolic and receptor‐mediated actions of glucose 68. While sucralose and other sweeteners may not reach a high enough concentration in the blood to activate the receptors on islets *in vivo*, sweeteners like sucralose provide a useful means to differentiate between receptor‐mediated and metabolic activities of glucose via the sweet receptor on β cells. In support of the importance of understanding this signaling, recent work suggests that sucralose or glucose signaling through taste receptors can impact intracellular metabolism resulting in increased ATP concentrations 68. The mechanism that links this signaling to the sucralose‐induced elevation of cAMP and calcium and how S6 becomes phosphorylated and what role its activation plays in beta cells downstream of taste receptor signaling remains to be investigated.

## Conclusions

We have discovered an unexpected role for a nonmetabolizable sweetener, sucralose, in activation of an ERK1/2‐S6 signaling axis. This signaling cascade is independent of mTOR and so far our data do not confirm that RSK plays a role in S6 phosphorylation in this case. These findings should serve to invigorate investigation into nutrient sensing from the plasma membrane in beta cells. This sensing involves multiple inputs leading to the activation of specific signaling pathways (amino acids, mTOR; sucralose, cAMP/S6; glucose, ERK1/2) that are usually difficult to deconvolve *in vivo*. Further understanding of these sensing events and the signaling pathways involved could impact human disease, given recent studies of sweet receptor T1R2 knockout mice which were conferred protection from hyperinsulinemia on high‐fat diets 69.

## Author contribution

MLG conceived the project. KM and MLG generated data. MLG, MAK, KM, and MHC analyzed and interpreted data. MLG, MAK, and MHC wrote and edited the manuscript.
